# A mixed-methods investigation into impact of motivation type on adherence and effect in iCBT for binge eating disorder

**DOI:** 10.1192/j.eurpsy.2023.989

**Published:** 2023-07-19

**Authors:** T. T. Holmberg, M. Sainte-Marie, E. K. Jensen, E. Runge, J. Linnet, M. B. Lichtenstein, K. Tarp

**Affiliations:** 1 Center for Digital Psychiatry, Region of Southern Denmark; 2 Research and Innovation Organisation, University of Southern Denmark; 3 Department of Occupational and Environmental Medicine, Odense University Hospital; 4 Department of Clinical Research, Faculty of Health Sciences, University of Southern Denmark, Odense C, Denmark

## Abstract

**Introduction:**

Motivation is an important factor in therapy and potentially even more so in an online setting. Earlier research shows that more autonomously motivated patients have better outcomes and completion rates than more controlled motivated patients´. However, little is known about how motivation type influences treatment effect in an online setting and in patients with binge eating disorder specifically.

**Objectives:**

This study set out to investigate how motivation type as per the Self-Determination Theory would affect treatment adherence and effect in a sample of 148 patients, undergoing an Internet-based Cognitive Behavioral Therapy (iCBT) for BED.

**Methods:**

The study was mixed-methods. A sample of 148 patients gave two written qualitative statements regarding their motivation for seeking treatment and reasons for choosing online therapy

The statements were transformed into quantitative units via the condensation method. The themes were categorized according to the model by Ryan and Deci based on level of autonomy and perceived locus of causality.

This was compared with completion rate and outcomes on eating disorder symptomatology. Completion was designated into three groups. Low adherers - less than six sessions (n=54), high adherers – between 7 and 10 sessions (n =56) and full adherers - 10 session plus follow up (n=37).

The effect of the treatment was measured via the Eating Disorder Examination Questionnaire (EDEQ) and Binge Eating Disorder Questionnaire (BEDQ).

**Results:**

**Table 1:** shows the distribution of patients’ motivational types regarding therapy aims

***Controlled***	***→***	***Autonomous***			
Motivational type:	*Introjection*	*Introjection*	*Identification*	*Integration*	
Patient motivation:	*Shame*	*Weight loss*	*Psychological* *stress*	*Insight*	*In all*
In all	25	25	50	48	148

**Table 2:** shows the distribution of patients´motivational types regarding online treatment

**Controlled**	**→**	**Autonomous**			
Motivational type:	*Introjection*	*Introjection*	*Identification*	*Integration*	
Patient motivation:	External	Avoidance	Convenience	*Reflection*	*In All*
In all	31	21	81	15	148

**Table 3:** shows the results from morivational types in each setting on BEDQ and EDEQ scores. No significant correlation was found.

Therapy Aims	BEDQ	0.92
	EDEQ	0.51
Why Online Therapy	BEDQ	0.99
	EDEQ	0.23

**Conclusions:**

Perceived locus of causality and level of autonomy, did not affect level of adherence or outcome of treatment in either setting. This unexpected result may suggest that internet-based therapy is less dependent on motivation types, when comparing with face-to-face treatment.

**Disclosure of Interest:**

None Declared

